# Characterisation of the Urinary Metabolic Profile of Liver Fluke-Associated Cholangiocarcinoma

**DOI:** 10.1016/j.jceh.2019.06.005

**Published:** 2019-07-31

**Authors:** Munirah Alsaleh, Paiboon Sithithaworn, Narong Khuntikeo, Watcharin Loilome, Puangrat Yongvanit, Nittaya Chamadol, Thomas Hughes, Thomas O'Connor, Ross H. Andrews, Elaine Holmes, Simon D. Taylor-Robinson

**Affiliations:** ∗ Division of Surgery and Cancer, Imperial College London, London, United Kingdom; † Cholangiocarcinoma Research Centre, Faculty of Medicine, Khon Kaen University, Khon Kaen 40002, Thailand

**Keywords:** bile duct cancer, mass spectrometry, metabonomics, *Opisthorchis viverrini*, acetaminophen, APAP, carnitine palmitoyltransferase 1, CPT1, carnitine palmitoyltransferase 2, CPT2, carnitine/acylcarnitine translocase, CACT, cholangiocarcinoma, CCA, cholangiocarcinoma screening and care program, CASCAP, data-dependent acquisition, DDA, electrospray ionisation, ESI, hypoxanthine-guanine phosphoribosyltransferase, HPRT, hypoxanthine phosphoribosyltransferase 1, HPRT1, orthogonal projections to latent structures discriminant analysis, OPLS-DA, primary biliary cholangitis, PBC, principal component analysis, PCA, periductal fibrosis, PDF, periportal fibrosis, PPF, primary sclerosing cholangitis, PSC, reversed-phase ultra-performance liquid-chromatography mass spectrometry, RP-UPLC-MS, ultra-performance liquid chromatography mass spectrometry, UPLC-MS, variable importance in projection, VIP

## Abstract

**Background:**

Human infection with *Opisthorchis viverrini*, a carcinogenic liver fluke inhabiting the biliary tree, is endemic in Southeast Asia. Chronic infection is associated with a fatal complication, cholangiocarcinoma (CCA), a late-presenting and aggressive malignancy. Currently, annual mortality rates from CCA mirror trends in incidence, due in part to limited availability of efficient prognostic and early diagnostic biomarkers. With ability to detect thousands of urinary metabolites using metabonomics, the urine metabolome holds great potential in providing an insight into system-level alterations in carcinogenesis and in identifying metabolic markers altered in response to disturbed homoeostasis.

**Methods:**

Global molecular profiling using reversed-phase ultraperformance liquid chromatography mass spectrometry was utilised to acquire the urinary spectral profile of 137 Thai subjects (48 at high risk of infection, 41 with *O. viverrini* infection, 34 periportal fibrosis and 14 CCA) from Khon Kaen, Thailand.

**Results:**

Multivariate statistical analysis identified perturbation in several molecular classes related to purine metabolism and lipid metabolism in the CCA urine metabolome. These markers mainly reflect changes in energy metabolism to support proliferation (increased fatty acid oxidation and purine recycling), DNA methylation and hepatic injury.

**Conclusions:**

Several metabolites of biological interest were discovered from this proof-of-principle dataset. Augmenting these findings is essential to accelerate the development of urinary metabolic markers in CCA.

The year 2015 marked 100 years of the human opisthorchiasis discovery in Thailand, and despite availability of effective treatments and control programs, which have been in place for decades, *Opisthorchis viverrini* and/or *Clonorchis sinensis* remain endemic in the countries of the Lower Mekong Basin, where the practice of raw cyprinid (scaly) fish consumption leads to high prevalence of parasite infection.^1^ It is reported that up to 20,000 new cases with liver fluke–induced cholangiocarcinoma (CCA) are detected annually in Thailand alone.^2^ Khuntikeo *et al*^3^ also reported that the *O. viverrini* infection landscape is beginning to shift among school children as a result of school education and community control programmes in Thailand. They predicted that CCA prevalence will decline within the next decade. The authors hoped that their data will motivate their colleagues in other countries of the Mekong Basin, such as the Lao Peoples’ Democratic Republic, Cambodia and Vietnam, to adapt similar strategies to impact the disease.^2^

To date, liver fluke–induced CCA incidence is still alarmingly high and will likely kill hundreds of thousands of people in the coming decades, affecting the poorest of the poor. There is an urgent, unmet demand for the development of noninvasive, efficient biomarkers that can hold clinical potential for timely diagnosis of *O. viverrini*–related hepatobiliary diseases.^1^ Human metabolic profiling studies of biofluids from CCA patients have identified a number of altered metabolites related to several metabolic pathways implicating changes in hepatic function, lipid metabolism and bile acid metabolism. No publication to date has examined the urinary metabolic profile of liver fluke–induced CCA, but other urinary markers have been explored.^4^ Urine contains a wealth of information, as small metabolites (<1000 Da) pass through the renal glomerulus as an ultrafiltrate of the blood. These metabolites provide a metabotype or a metabolic ‘fingerprint’ pattern of the host physiological status.

We aimed to investigate CCA metabolic signatures in a Thai population that distinguish early CCA development from patients with periductal fibrosis, caused by *O. viverrini* infection, distinct from those with parasitic infection, but without ductal fibrosis. Global profiling using ultraperformance liquid chromatography mass spectrometry (UPLC-MS) offers the portent of urinary metabolite discrimination and was the methodology chosen to investigate whether urine MS spectral profiles from patients with *O. viverrini*–induced CCA are different from nonmalignant control populations.

## Methodology

### Sample Collection

Study samples were collected from the Isaan, who are an ethnic community native to Northeastern Thailand. Raw fish dishes containing *O. viverrini* parasites are distinctive to their cultural cuisine. Patients with CCA were recruited consecutively from inpatient populations in Khon Kaen Hospital, Khon Kaen, Thailand. Malignant strictures were diagnosed by computed tomography or magnetic resonance imaging and further confirmed by histology at surgical operation. Spot urine samples were collected from each participant before undergoing any treatment. The control groups were collected from the cholangiocarcinoma screening and care program (CASCAP) field screening programme in endemic Northeastern region.^5^ Isaan Thais of ≥ 40 years old who were known to have any of the following were eligible to be included in the study: (i) ever been infected with liver fluke; (ii) ever been treated for liver fluke and/or (iii) ever have eaten uncooked freshwater fish with scales. Once consent had been obtained, participants were enrolled in the CASCAP program and baseline demographic information, hepatic ultrasound examination and parasitological fecal test for *O. viverrini* eggs were collected.

Samples were grouped into 3 subsets: (1) participants positive for *O. viverrini* eggs in stool and with normal ultrasonography; (2) participants positive for parasite-induced periportal fibrosis (PPF) and (3) participants at high risk of opisthorchiasis (negative ultrasound findings and stool test). Participants were categorised according to findings from fecal and ultrasound examination.^6^

### Ultrasound Criteria

**Figure 1:**
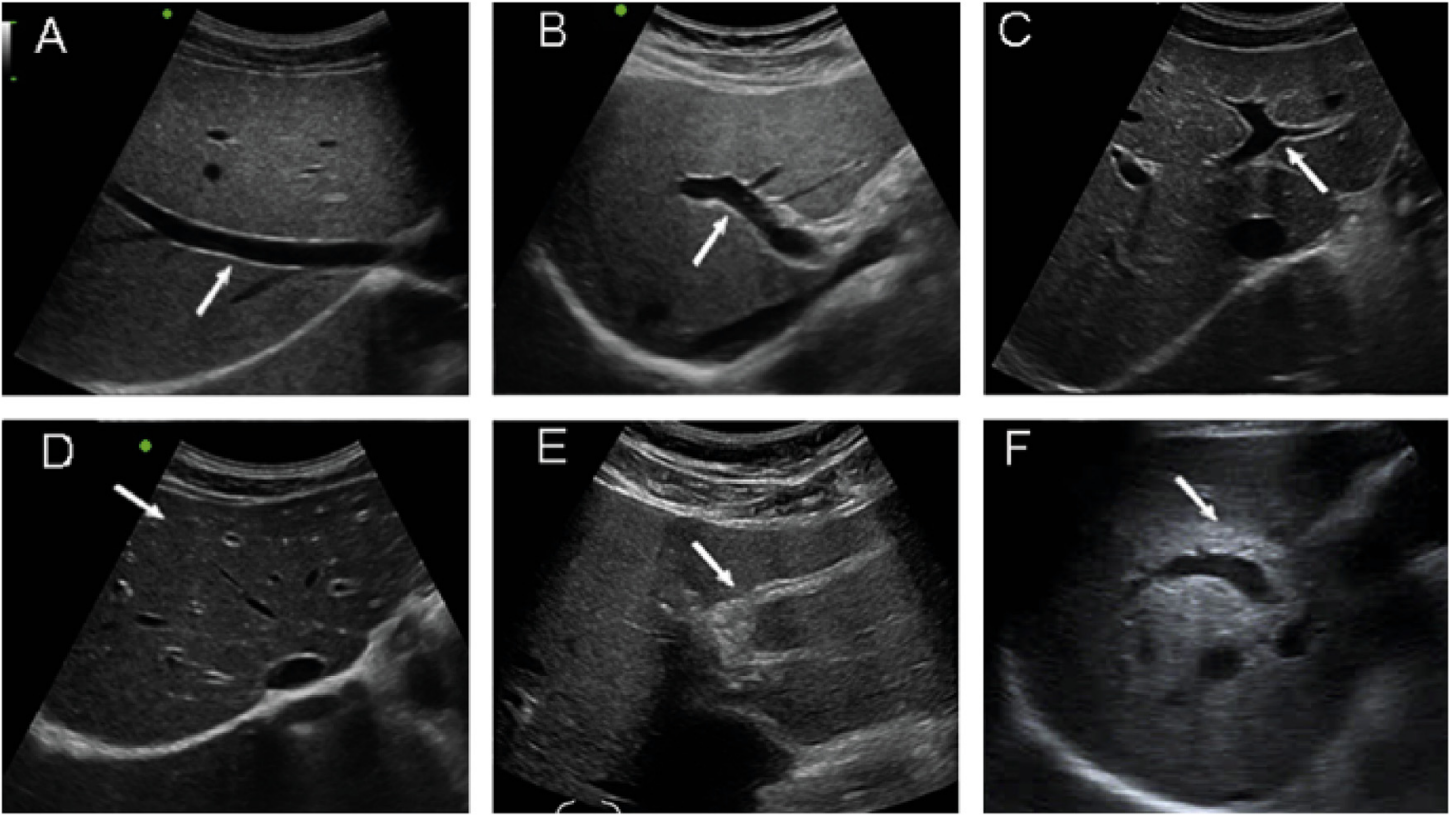
Ultrasound typing of periductal fibrosis (PDF). Normal echo pattern of the liver (A–C) with arrow pointing at (A) the right hepatic vein, (B) right portal vein and (C) left portal vein. (D) PDF+ shows a ‘starry sky appearance’, with bright echogenic dots along branches of portal vein (arrow); (E) PDF++ shows ring echoes around vessels in cross-section, pipe-stems parallel with portal vein (arrow) and (F) PDF+++ shows an echogenic ruff around the portal bifurcation and main stem; the main portal vein vessels show wall thickening (arrow). Figure reproduced with permission of the copyright owner.^6^

A mobile, high-resolution ultrasound was used to assess hepatobiliary abnormalities. The liver normal sonographic appearance is typically homogeneous with visible portal and hepatic veins. Normal hepatic arteries and bile ducts are usually nonechoic because of thin walls and small size. In periductal fibrosis (PDF), fibrotic thickening of the bile duct wall in the periportal space shows up as increased periportal echoes. Histological confirmation of periductal fibrosis was documented. The increase periportal echo which represents periductal fibrosis was measured and classified according to the method recommended by the World Health Organization to diagnose schistosomiasis-related PPF.^7^ Ultrasound findings were classified into the following:

0 = ultrasound negative;

1 = ultrasound positive grade 1 (PDF 1+);

2 = ultrasound positive grade 2 (PDF++);

3 = ultrasound positive grade 3 (PDF++); (Figure 1);

4 = suspected CCA.

### Ethical Considerations

Urine samples were obtained from each consented subject and stored at −80 °C in the Specimen Bank of the Liver Fluke and Cholangiocarcinoma Research Center, Faculty of Medicine, Khon Kaen University, Khon Kaen, Thailand. The study was approved by the Human Research Ethics Committee (reference no. HE571283 and HE521209). Written informed consent was obtained from each participant.

### Sample Transport and Preparation

Samples were transported with a courier service from Khon Kaen University and kept frozen on dry ice until received and frozen at −80 °C at the Liver Unit in St. Mary's Hospital in London, UK. Before analysis, frozen urine samples were left to thaw at room temperature, then vortex mixed and centrifuged at 16089 g for 10 min. After randomisation, an aliquot of 250 μL of each sample were transferred into 96-well 350 μL plates with cap mats from Waters Corporation (Milford, MA, USA). Prepared samples were kept in a 0–4 °C fridge and then at 4 °C throughout the analysis in an autosampler.

### Quality Control

Quality control (QC) samples were prepared by pooling 50 μL of each urine sample into a Falcon tube (Sigma-Aldrich, Dorset, UK). A 200-μL aliquot was then transferred to an analytical MS well plate to enable acquisition of a QC spectrum in every 10 samples.

### Chromatographic Conditions

The sample spectra were acquired using an ACQUITY™ UPLC system (Waters Ltd. Elstree, UK), coupled to a LCT Premier™ mass spectrometer (Waters MS Technologies, Ltd., Manchester, UK). Reversed-phase (RP)-UPLC-MS was performed with electrospray ionisation (ESI) in both positive and negative modes. The conditions were optimised using the QC samples in terms of peak shape, reproducibility and retention time.

### Tandem Mass Spectrometry

Tandem mass spectrometry (MS/MS) analysis was performed using a quadrupole time-of-flight (TOF) Premier™ instrument (Waters MS Technologies, Manchester, UK). Collision-induced dissociation (CID) experiments of the QC sample were performed for structural elucidation of detected ions in each ionisation mode. This was conducted subsequent to the original profiling run to save time and limit analytical variations in retention time and performance that can occur when returning to the instrument for CID analysis.

Two complementary MS/MS acquisition modes were used to ensure sufficient MS/MS coverage of ions of interest, data-dependent acquisition (DDA) and acquisition with no precursor ion selection or data-independent acquisition (MS^E^). The DDA experiment was set to switch automatically from MS to MS/MS mode using data-dependent criteria. It triggered MS/MS on the most abundant ions in each MS scan and provided fragments specifically attributed to the precursor ion. In MS^E^ mode, eluting peaks were subjected to both high and low collision energies in the collision cell of the mass spectrometer, with no prior precursor ion selection.^8^

### Metabolite Assignment Verification

Molecular mass, retention time and fragmentation spectrum of the discriminant features were compared against online spectral libraries such human metabolome database (HMDB) (www.hmdb.ca)^9^ and metabolite and chemical entity database (METLIN) (https://metlin.scripps.edu).^10^ Metabolites were classified as either (a) identified compounds confirmed with authentic standards; (b) putatively annotated compounds (such as those based on fragmentation pattern and/or spectral similarity with spectral databases); (c) putatively identified to match a certain chemical class (such as those based on spectral similarity to known compounds of a chemical class) or (d) unknown compounds.

### Preprocessing

Raw LC-MS data files were converted to compact disc video (CDV) format by MassLynx™ version 4.1 application manager (Waters Corporation, Milford, MA, USA) and then imported into R Project version 3.1.0 (The R Foundation for Statistical Computing, 2014) for preprocessing using XCMS package version 2.14 (Bioconductor). Computational scripts written in-house were applied to (1) filter and identify peaks; (2) correct for retention time drift; (3) match peaks across samples and (4) fill in missing peaks.

### Statistical Analysis

SIMCA-P+ version 13.0.2 (Umetrics, Umeå, Sweden) was used for multivariate statistical analysis of the processed data. Initial analysis was performed using unsupervised principal component analysis (PCA) to explore variation in the dataset and examine clustering patterns or trends in the dataset, based on metabolic profile similarities or differences. Following PCA, orthogonal projections to latent structures discriminant analysis (OPLS-DA) was performed to maximise separation between predefined sample classes to view discriminatory features. Feature selection was based on variable importance in projection (VIP) coefficients, which allow X-variables to be classified according to their explanatory power of Y (class information). Features with high VIP value, larger than 1, were found to be the most relevant for explaining Y class information. The top 30 features were selected and identified for each model.

Validating multivariate models is essential to avoid overfitting data. Model statistics, R^2^X, Q^2^Y, permutation test and cross validated (CV)-analysis of variance (ANOVA) p-value, were used to evaluate model robustness. Permutation testing (with 100 permutations) was calculated for every OPLS-DA model using SIMCA-P+ version 13.0.2 (Umetrics, Umeå, Sweden). Univariate significance tests were then performed on selected features using ANOVA with *post hoc* tests (Tukey's honestly significant difference (HSD)) which are designed to account for multiple comparisons. Significant features were presented graphically as box and whisker plots.

### Correlation with Hierarchical Clustering Order

R Project version 3.1.0 (The R Foundation for Statistical Computing, 2014) using corrplot package version 0.77 (CRAN) was used to perform hierarchical cluster analysis of Spearman's correlation coefficient matrix. Cluster analysis was used to investigate correlations among identified biochemical components. The correlation matrix was represented as a heatmap with rows and columns ordered according to hierarchical clustering analysis. Positively and negatively correlated analytes were displayed in blue and red colours, respectively. A circle was used to represent correlations between pairs of compounds. The circle diameter and colour intensity were proportional to correlation coefficients and indicated statistically significant correlations (*<* 0.05). The circle diameter and colour intensity were proportional to the correlation coefficients. Nonsignificant correlations were represented as an X.

## Results

### Urine RP-UPLC-MS Chromatogram

**Figure 2:**
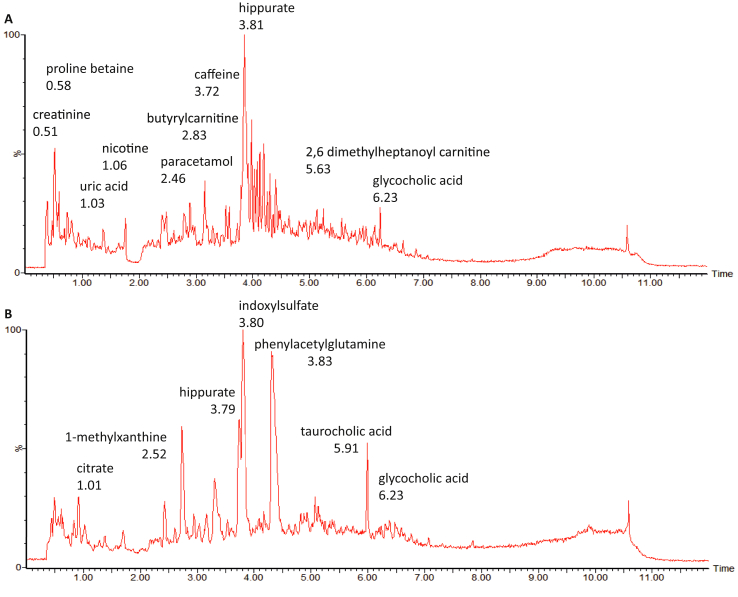
Example of compounds detected in the nontargeted liquid chromatography mass spectrometry. (A) positive and (B) negative electrospray ionisation mode.

A nontargeted metabonomics approach using LC-MS was applied to fingerprint the urinary metabolome of CCA patients in comparison to controls. A total of 1,993 and 1,224 features were detected in the positive and negative ESI modes, respectively. Given that urine is a complex biological fluid, those features can represent metabolic breakdown products from dietary intake, drugs use, endogenous waste metabolites, gut microbiota by-products and environmental contaminants. Figure 2 shows an example of detected compounds in LC-MS–positive (A) and LC-MS–negative (B) ionisation mode.

### Demographics, Clinical Data and Cohort Description

A total of 137 Thai participants were recruited to form the training set. CCA patients (n = 14) were older compared to controls and comprised mostly males (71.4%). Similarly, male gender was greater in all control groups: high risk (75%), *O. viverrini* (63%) and PPF (56%). The majority of CCA patients presented with intrahepatic tumours, but no additional information regarding the patient's blood biochemistry tests, tumour stage/grade, liver function and comorbidities was available. Of 34 patients, eight participants had active opisthorchiasis, 27 had moderate fibrosis (PPF+) and seven were diagnosed with PPF++. The opisthorchiasis group egg count ranged from 6 to 880 eggs per gram of feces. Patient characteristics are summarised in Table 1.

**Table 1:** Patient Demographics.

Characteristic	Cholangiocarcinoma	High risk	Opisthorchiasis	Periductal fibrosis
**Participants**, n	14	48	41	34
**Age**, mean, (range), y	60.3 (29–77)	58.6 (44–80)	58.9 (40–86)	51.1 (40–74)
**Male**, %	71.4	75.0	63.4	56.9
**Smoking**, (n)				
Smokers	–	10	17	12
Nonsmokers	–	26	19	17
Not available	14	12	5	5
**Tumour location**				
Intrahepatic	6			
Hilar	2			
Distal	1	–	–	–
Gallbladder	3			
Unspecified	2			
***O. viverrini* FECT**, *n*				
0		48	0	26
1–50		0	17	5
51–100	–	0	11	2
>100		0	13	1
**Fibrosis severity**, n				
PPF+	–	–	–	27
PPF++				7

PPF, periportal fibrosis; FECT, formalin ether concentration technique.

### Global Overview of Study Samples

**Figure 3:**
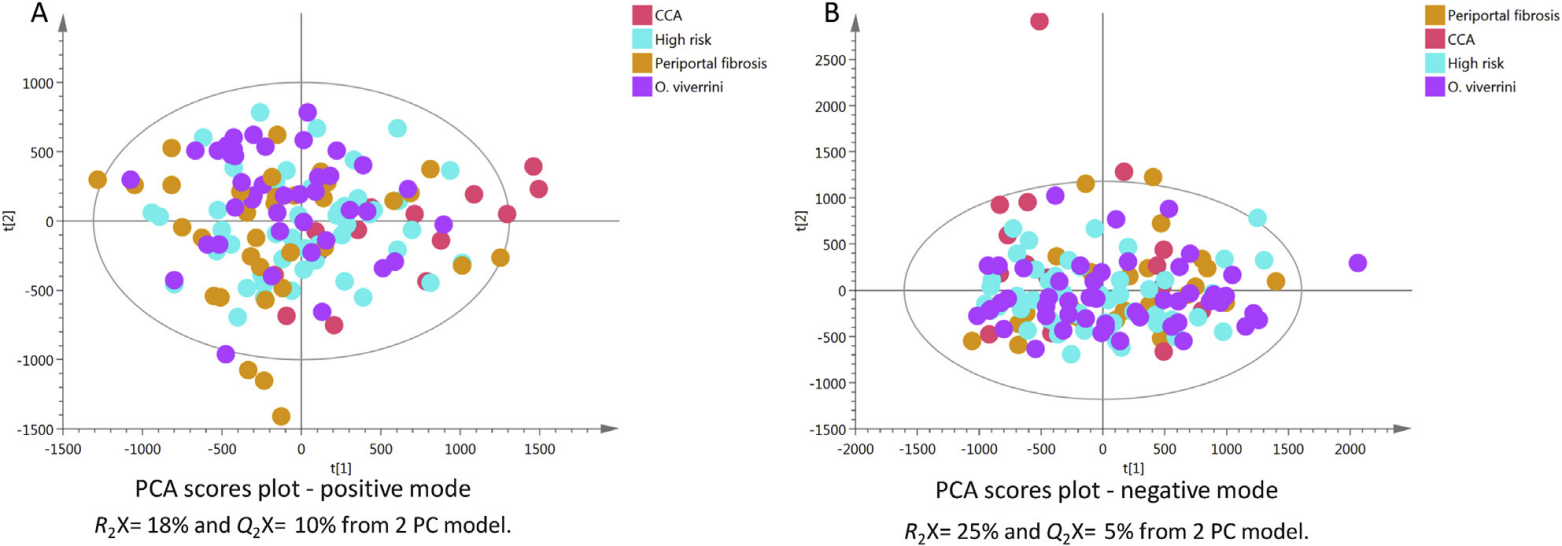
PCA scores plots of test samples derived from UPLC-MS urinary data (*n* = 137 participants) for (A) positive and (B) negative ion mode. CCA, cholangiocarcinoma; PCA, principal component analysis; UPLC-MS, ultraperformance liquid chomatography mass spectrometry.

An initial PCA model was calculated from all samples acquired using positive (Figure 3a) and negative (Figure 3b) modes to provide a global overview of the data. No clear clustering pattern was observed between groups (high risk, *O. viverrini–*infected, PPF and CCA), which might be because of high intraclass variability. The percentage of explained variance was 18% and 25% for positive and negative modes, respectively.

### Environmental Influences

#### Drug Exposure

Paracetamol or acetaminophen (APAP) is a widely used nonprescription analgesic drug. Following initial data exploration, peaks corresponding to APAP urinary metabolism products were found to be discriminant between cancer patients and control groups. Features with correlation coefficients higher than 0.8 with the most predominant APAP product were examined and excluded in both positive and negative ionisation modes. APAP (M+H, *m/z* 152.071) is mainly metabolised via conjugation and renally excreted as APAP-glucuronide (M+H *m/z* 328.103, APAP-sulphate (M+H, *m/z* 232.028) and APAP-N-acetylcysteinylate (M−H, *m/z* 311.076).

The peaks of APAP and its urinary products were excluded to enable identification of biologically relevant features from endogenous metabolites.

#### Impact of Dietary Exposure

**Figure 4:**
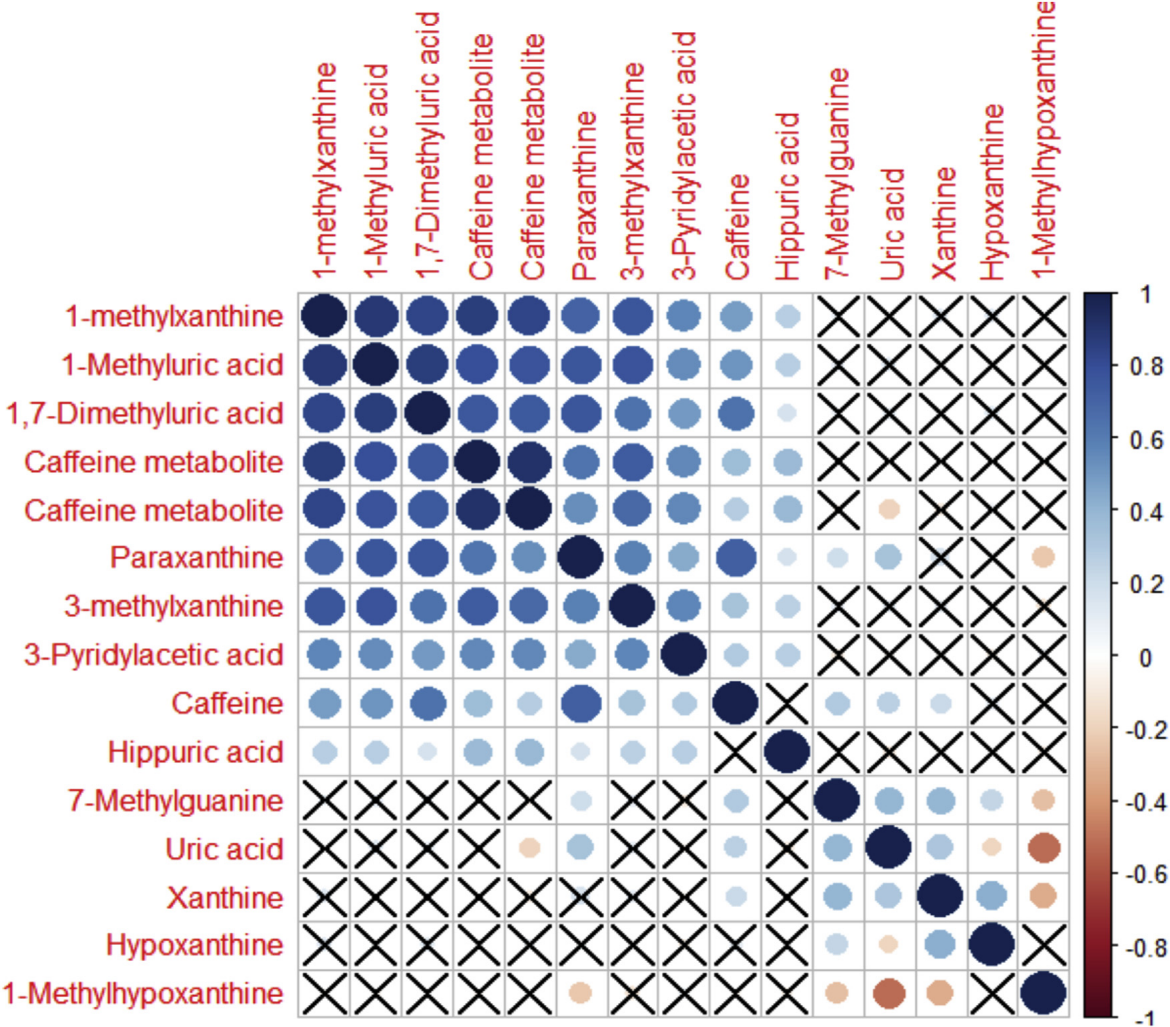
Heatmap of metabolites related to caffeine and purine metabolism. The blue circles represent positive correlations, whereas the red ones show the negative correlations. The larger the circle diameter, the higher the correlation (the X indicates no significant correlation, p > 0.05).

Dietary information from the 24-hr dietary recall regarding the intake of meat (pork, beef, chicken and fish), vegetables, herbs, tea and coffee was used to identify markers related to foods and dietary pattern. Difference in the detected metabolic profiles based on food intake was only pronounced between individuals who had reported to consume tea or coffee.^11^ Caffeine and its metabolites were predominant in the control groups. A high degree of correlation was observed among caffeine analytes, as shown in the heatmap (Figure 4). The heatmap also shows clear clustering between purine metabolites which are present in the caffeine pathway and purines, such as xanthine, hypoxanithne and uric acid, which are in the purine metabolism pathway.

#### Tobacco Smoking

Nicotine metabolites were found to be discriminant between the control and cancer groups. Therefore, a model using spectral data from smokers (*n* = 39) versus nonsmokers (*n* = 62) was calculated to examine the impact of smoking on the metabolic profiles. Nicotine metabolites (nicotine, hydroxy-cotinine and cotinine) were found in greater concentrations in urine samples from smokers and were detected in the positive mode, *Q*^2^Y = 35%. As most of smokers were males (3 females out of 39 smokers), gender-related metabolites were also found to influence the model. After excluding nicotine-related metabolites from the OPLS-DA model, the cross-validation statistics of the positive spectral data were not predictive (*R*^2^Y = 64%, *R*^2^X = 14% and *Q*^2^Y = 14%), possibly indicating mild tobacco exposure impact on the endogenous metabolic profile of the smokers.

#### Gender Effects

Gender difference is a known confounder in metabonomic studies. When first examined, the nicotine metabolite, hydroxy-nicotine, was the most discriminant feature between males and females (VIP 9.3). Therefore, nicotine-related metabolites were excluded to examine genuine gender-related differences. A separation trend between males and females (*Q*^2^Y = 31%) was observed only in the positive mode spectral data.

#### Cancer Patients vs. High-Risk Healthy Control Group

**Figure 5:**
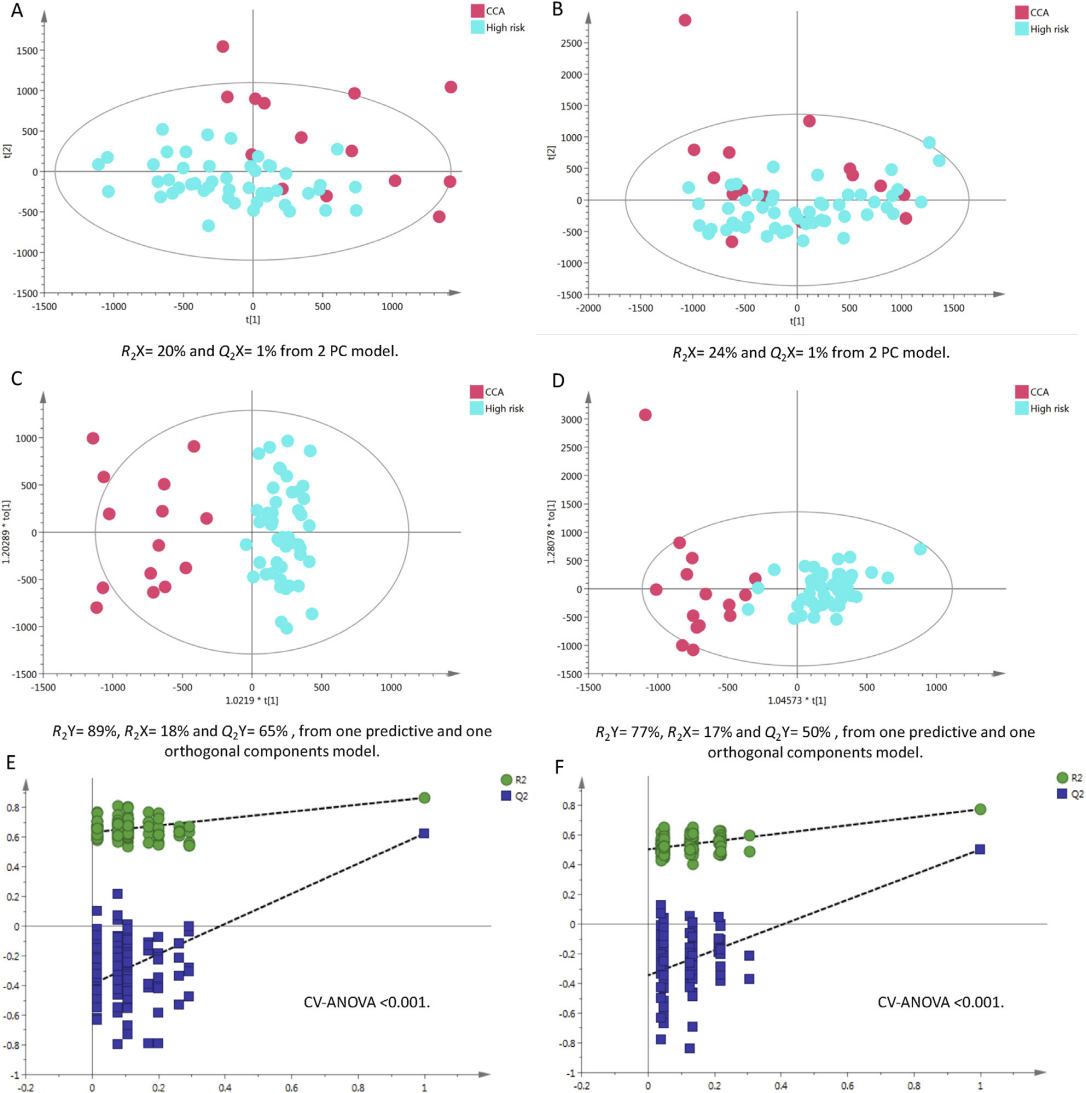
PCA scores plots for (A) positive and (B) negative ion mode data of CCA patients and healthy controls. OPLS-DA score plots showing group separation for both (C) positive and (D) negative ion mode data and the corresponding permutation tests for (E) positive and (F) negative ion mode data. CCA, cholangiocarcinoma; PCA, principal component analysis; OPLS-DA, orthogonal projections to latent structures discriminant analysis.

The PCA model of urine samples from high-risk Thai participants versus cancer patient samples showed a clustering trend between the two groups in positive, but not in negative modes, whereas OPLS-DA statistics showed enhanced separation in both modes with good reproducibility (Figure 5c, positive Q^2^Y = 65% and Figure 5d, negative Q^2^Y = 50%). A permutation test (Figure 5e&f, p-value <0.001) was used to evaluate the robustness of the OPLS-DA model and ensure that the Q^2^Y and R^2^Y values were not random or overfitted.

#### Cancer Patients vs. Opisthorchiasis Carriers

**Figure 6:**
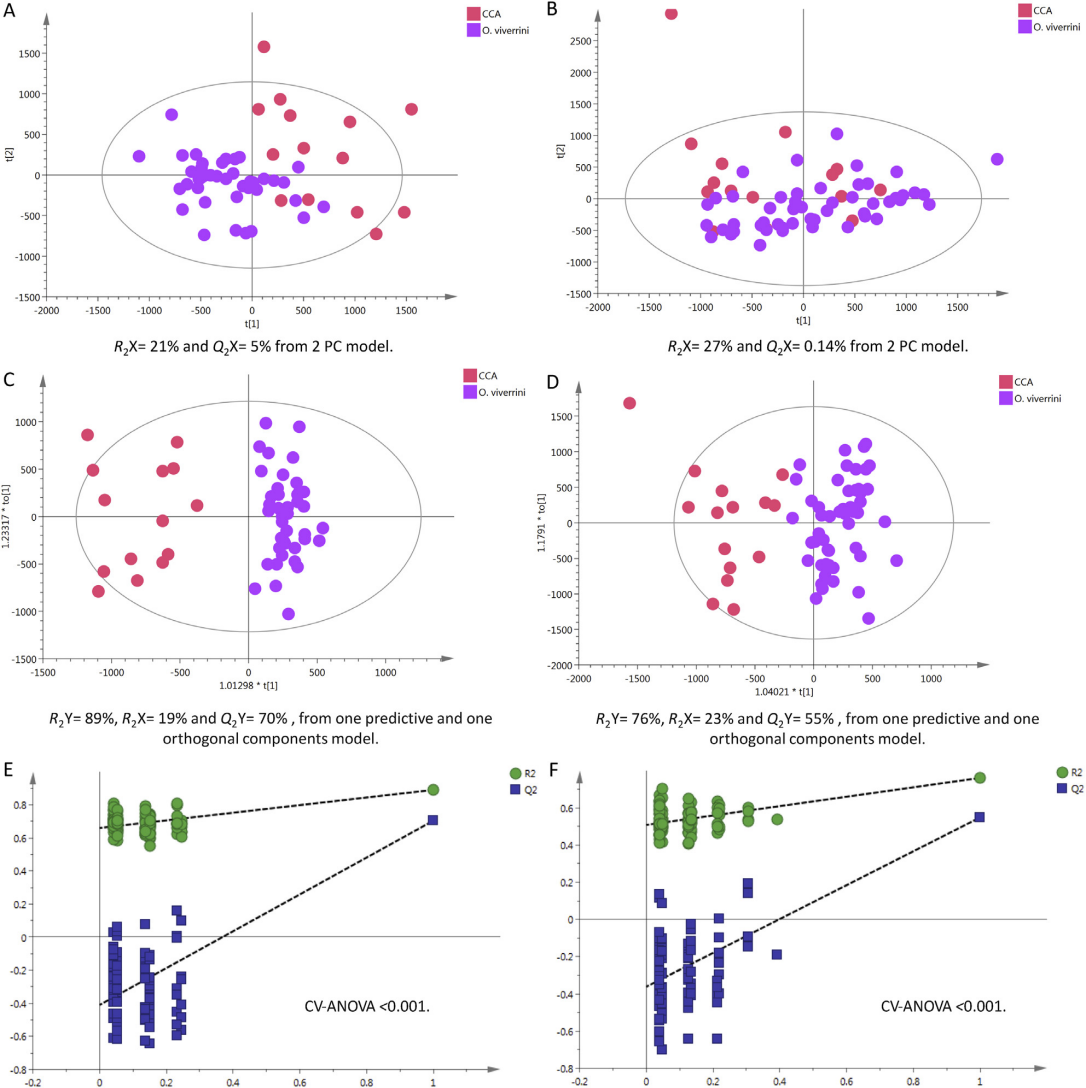
PCA scores plots for (A) positive and (B) negative ion mode data of CCA patients and *O. viverrini–*infected cases. OPLS-DA score plots showing group separation for both (C) positive and (D) negative ion mode data and the corresponding permutation tests for (E) positive and (F) negative ion mode data. CCA, cholangiocarcinoma; PCA, principal component analysis; OPLS-DA, orthogonal projections to latent structures discriminant analysis.

A clear clustering pattern was observed in the positive-mode PCA between CCA patients and *O. viverrini*–infected cases (Figure 6a). Supervised analysis showed high model predictive ability with *Q*^2^Y = 70% and 55% in positive (Figure 6c) and negative (Figure 6d) ionisation modes, respectively. The permutation validation plots assured that the OPLS-DA models were reliable as original points (right) were lower than the permuted points (left) and p < 0.001 (Figure 6e&f).

#### Cancer Patients vs. Subjects with PPF

**Figure 7:**
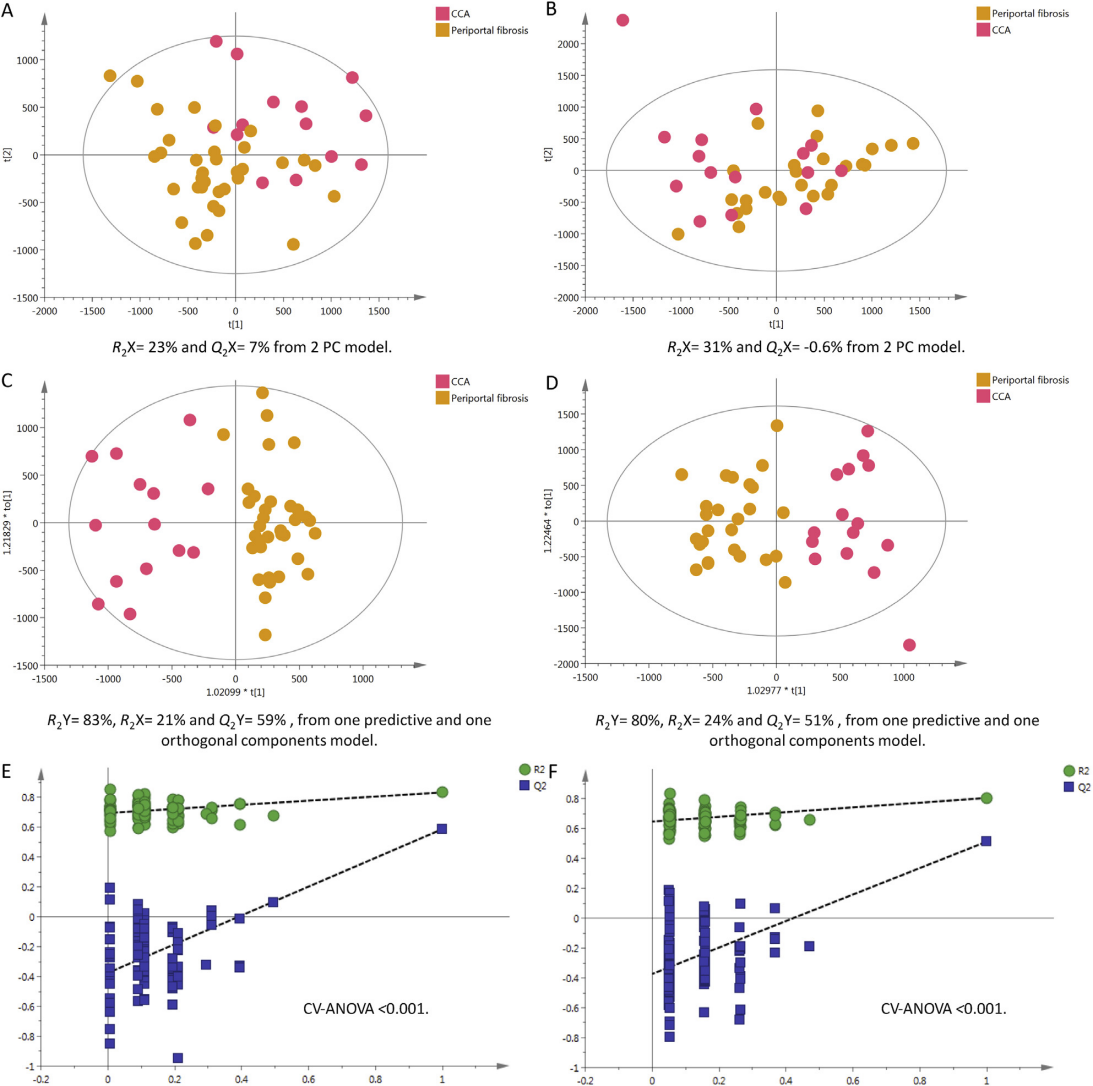
PCA scores plots for (A) positive and (B) negative ion mode data of CCA patients and periportal fibrosis cases. OPLS-DA score plots showing group separation for both (C) positive and (D) negative ion mode data and the corresponding permutation tests for (E) positive and (F) negative ion mode data. CCA, cholangiocarcinoma; OPLS-DA, orthogonal projections to latent structures discriminant analysis

Participants with PPF (fibrous tissue surrounding the hepatic portal vein and its branches), which usually results from chronic *O. viverrini* infection showed good separation from the CCA group. The positive mode statistics are R^2^Y = 83%, R^2^X = 21% and Q^2^Y = 59% and negative mode statistics are R^2^Y = 80%, R^2^X = 24% and Q^2^Y = 51%, as illustrated in (Figure 7c&d).

#### Metabolites Altered in CCA

**Figure 8:**
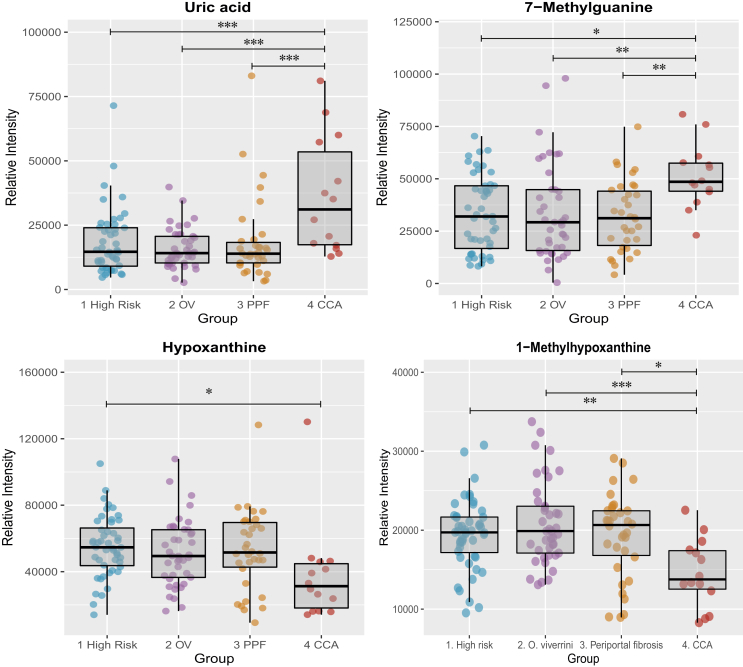
Box and whisker plots showing altered purine compounds. CCA, cholangiocarcinoma; OV, *Opisthorchis viverrini*; PPF, periductal fibrosis. ∗p 0.05, ∗∗p 0.01 and ∗∗∗p 0.001.

**Figure 9:**
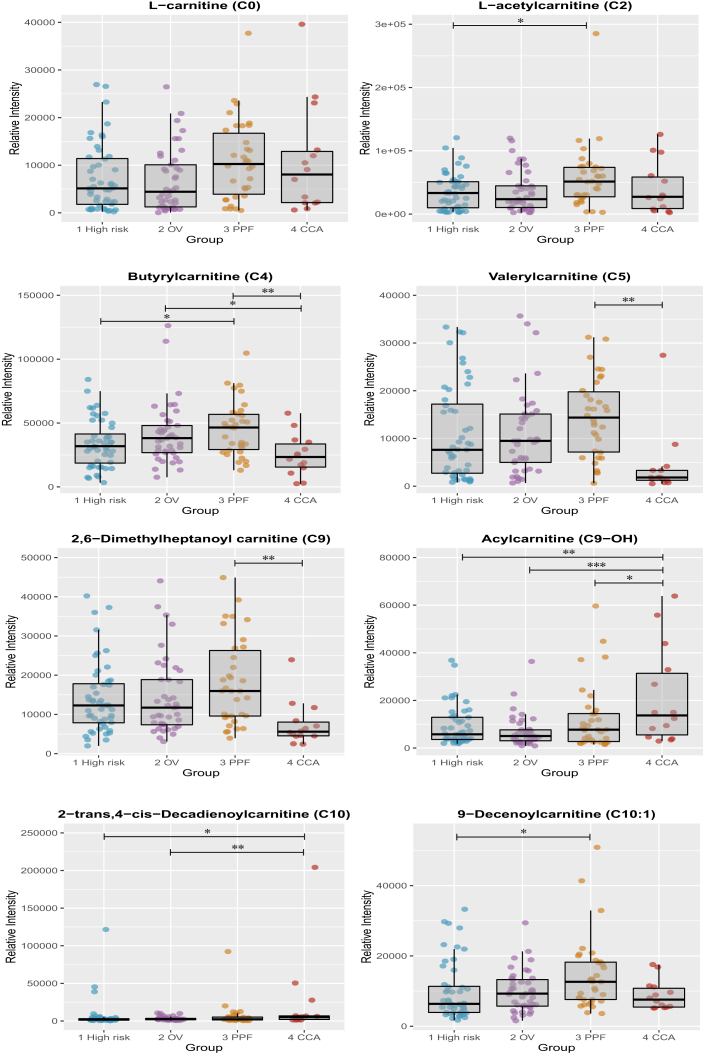
Box and whisker plots showing altered acylcarnitine compounds. CCA, cholangiocarcinoma; OV, *Opisthorchis viverrini*; PPF, periductal fibrosis. ∗p 0.05, ∗∗p 0.01 and ∗∗∗p 0.001.

The relative intensities of several compounds, among which are purines (Figure 8), carnitines (Figure 9), bile acids and steroid glucuronide species, were found to drive the separation between the CCA and control groups, which implicate changes in energy production, hepatic function, fatty acid metabolism and bile acid metabolism. Univariate analysis on those compounds is illustrated in the box and whisker plots below. The differential metabolites between the groups are listed in Table 2, Table 3 detected in ESI^+^ and ESI^*−*^, respectively.

**Table 2:** Discriminant Compounds Between Cholangiocarcinoma Patients and Controls—Positive Mode.

*m/z*	RT	Tentative assignment	Adduct	Mass error	HR vs CCA			OV vs CCA			PPF vs CCA			Identification^a^
					*P*-value^c^	FC^b^	VIP	*P*-value^c^	FC^b^	VIP	*P*-value^c^	FC^b^	VIP	
114.065	0.51	Creatinine	M+H	10	NS	1.04	4.4	NS	−1.13	5.2	NS	1.05	4.9	a
144.102	0.58	Proline betaine	M+H	0	<0.001	6.13	15.2	<0.001	7.29	14.6	<0.001	4.14	12	b
229.119	0.60	Hydroxyprolyl-proline	M+H	1	NS	1.02	1.7	NS	1.22	2.5	NS	1.23	3.2	b
204.125	0.83	l-Acetylcarnitine (C2)	M+H	9	NS	1.15	1.5	NS	1.14	0.6	NS	−1.38	3.7	a
169.036	1.03	Uric acid	M+H	2	<0.001	1.94	5.9	<0.001	2.25	5.8	<0.001	2.02	5.6	a
137.046	1.10	Hypoxanthine	M+H	1	0.043	−1.46	4.6	NS	−1.36	3.3	NS	−1.41	3.9	b
166.073	1.16	7-Methylguanine	M+H	3	0.013	1.53	4.9	0.020	1.55	4.0	0.009	1.72	4.5	b
229.155	2.23	Leucyl proline	M+H	1	0.050	−1.32	2.9	0.007	−1.41	3.2	0.014	−1.39	3.1	b
174.124	1.85	Unknown	–	–	0.013	2.61	4.2	0.061	2.12	3.5	0.061	2.16	3.2	d
188.07	2.51	Unknown	–	–	0.034	30.4	5.1	0.008	41.7	4.9	0.048	35.1	4.7	d
120.081	2.51	Phenylalanine	Fragment	–	NS	−1.23	3.8	NS	−1.14	2.9	NS	−1.30	3.9	b
151.062	2.59	1-Methylhypoxanthine	M+H	3	0.008	−1.33	2.6	<0.001	−1.42	3	0.014	−1.33	2.4	b
232.155	2.83	Butyrylcarnitine (C4)	M+H	2	NS	−1.35	3.4	0.050	−1.67	4.5	0.010	−1.83	5.4	a
173.081	2.83	Unknown	–	–	NS	3.58	2.8	NS	3.32	2.6	NS	3.03	2.3	d
180.088	2.87	Unknown	–	–	0.020	−1.26	3	0.021	−1.28	3	0.04	−1.23	3.2	d
126.092	3.00	Unknown	–	–	0.011	−1.68	3.9	0.009	−1.56	3.3	0.002	0.58	4.5	d
188.071	3.16	3-Amino-2-naphthoicacid	M+H	2	NS	−1.12	4.4	NS	−1.0	4	NS	−1.16	4.5	b
211.058	3.31	Unknown	–	–	NS	2.15	2.7	NS	1.98	2.3	NS	2.11	2.2	d
246.17	3.62	Valerylcarnitine (C5)	M+H	0	NS	−2.61	2.8	NS	−2.63	3.0	0.002	−3.44	3.5	a
318.191	3.78	Acylcarnitine (C9–OH)	M+H	–	0.002	2.33	4.6	<0.001	3.1	4.4	NS	1.82	3.7	b
397.072	3.81	Unknown	–	–	NS	1.59	3.9	0.029	1.96	4.4	0.008	2.28	4.2	d
105.033	3.81	Hippurate	Fragment	–	NS	−1.33	3.7	NS	−1.41	3.3	NS	−1.22	1.8	a
265.118	3.85	Unknown	–	–	NS	−1.36	3.9	0.013	−1.65	5.9	0.044	−1.51	5.2	b
304.212	4.47	Unidentified acylcarnitine	–	–	NS	−2.57	1.8	NS	−2.31	1.8	0.002	−3.90	3.2	c
312.217	5.16	2-trans,4-cis-decadienoylcarnitine	(C10:2)	M+H	2	NS	3.35	4.50.018	6.68	5.3	NS	3.43	3.8	b
300.218	5.22	Unidentified acylcarnitine	–	–	NS	−1.06	2.2	NS	−1.01	1.8	NS	−1.38	3.4	c
302.233	5.61	2,6 Dimethylheptanoylcarnitine (C9:0)		NS	−1.87	3	NS	−1.93	2.6	0.003	–	2.43	4	b
314.233	5.78	Acylcarnitine (C10:1)	M+H	–	NS	−1.13	1.8	NS	−1.12	1.3	0.050	−1.64	2.9	c
328.248	6.11	Acylcarnitine (C10:2-OH)	M+H	0	NS	−1.35	1.7	NS	−1.44	1.9	NS	−2.31	3.6	c
430.296	6.24	Glycocholic acid	M+Na	2	<0.001	31.2	2.7	<0.001	44.7	2.6	<0.001	53.8	2.5	a

CCA, cholangiocarcinoma; HR, high risk; FC, fold change; OV, *Opisthorchis viverrini*; PPF, periportal fibrosis; RT, retention time; VIP, variable importance in projection score.

a Level of metabolite identification: (a) Identified compound; (b) putatively annotated compound; (c) putatively characterised compound class; and (d) Unknown.

b Negative FC value indicates down-regulated metabolites in cholangiocarcinoma.

c NS = not significant (*p* > 0.05).

**Table 3:** Discriminant Compounds Between Cholangiocarcinoma Patients and Controls—Negative Mode.

*m/z*	RT	Tentative assignment	Adduct	Mass error	HR vs CCA			OV vs CCA			PPF vs CCA			Identification^a^
					*P*-value^c^	FC^b^	VIP	*P*-value^c^	FC^b^	VIP	*P*-value^c^	FC^b^	VIP	
191.018	0.97	Isocitrate	M-H	9	NS	−1.10	2.1	NS	−1.01	1.5	NS	−1.15	1.2	b
167.02	1.03	Uric acid	M-H	6	0.005	2.16	5.5	0.011	1.92	4.9	0.023	1.93	4.6	a
191.019	1.06	Citrate	M-H	3	NS	−1.08	1.6	NS	1.37	2.1	NS	−1.00	0.9	a
260.022	1.78	Unknown	–	–	0.024	−1.26	2.1	NS	−1.25	−1.6	NS	−1.24	1.8	d
181.035	2.38	*N*-methyluric acid	M-H	9	NS	−1.89	1.9	NS	−2.02	1.8	0.008	−2.26	2.1	c
216.98	2.97	*N*-Sulfooxybenzoate	M-H	5	NS	1.05	1.4	NS	−1.20	2.2	NS	−1.08	1.9	b
195.051	3.04	*N*-dimethyluric acid	M-H	6	NS	−1.06	1.9	NS	−2.84	1.7	0.006	−3.98	2.3	c
188.985	3.15	Pyrocatechol sulphate	M-H	6	NS	−1.64	2.9	NS	−1.74	3.1	NS	−1.61	3.6	b
194.045	3.16	4-Hydroxyhippurate	M-H	4	NS	−1.45	2.1	NS	−1.56	1.90	NS	−1.01	0.7	b
242.012	3.16	Sulphated compound	–	–	NS	−3.38	1.4	NS	−5.84	3.60	NS	−3.20	1.6	c
203.001	3.74	O-methoxycatechol-O-sulphate	M-H	5	0.023	−2.00	2.8	<0.001	−2.75	3.37	0.008	−2.27	3.2	b
178.049	3.80	Hippurate	M-H	11	NS	−0.96	5.3	NS	1.11	2.04	NS	1.29	2.6	a
263.102	3.83	Phenylacetylglutamine	M-H	6	<0.001	1.66	8.5	0.006	1.53	7.68	0.007	1.55	7.2	b
212.001	3.86	Indoxylsulphate	M-H	10	NS	−1.30	10.7	NS	−1.32	6.00	NS	−1.52	10.2	a
245.012	3.93	Vanillin 4-sulphate	M-H	2	NS	−4.27	2.8	NS	−4.82	3.15	NS	−3.38	2.0	b
230.012	4.17	Benzeneacetamide-4-O-sulphate	–	–	NS	−2.24	1.3	NS	−3.71	2.81	NS	−2.30	1.6	c
187.005	4.41	p-Cresol sulphate	M-H	10	NS	−1.06	8.2	NS	−1.29	15.67	NS	−1.32	14.7	b
345.154	4.88	Unknown	–	–	0.016	4.58	4.4	0.001	7.45	4.17	0.012	4.41	3.5	d
541.265	5.14	Cortolone-3-glucuronide	M-H	0	0.003	−1.91	2.0	0.005	−1.88	1.71	0.002	−1.97	2.1	b
539.249	5.42	Tetrahydroaldosterone-3-glucuronide	M-H	1	<0.001	−2.13	2.1	<0.001	−2.12	1.88	0.001	−2.14	2.4	b
481.243	5.53	11-beta-Hydroxyandrosterone-3-glucuronide	M-H	2	<0.001	−2.30	2.0	0.004	−2.06	1.57	0.012	−1.98	1.8	b
331.175	5.98	Steroid glucuronide	–	–	0.018	−1.75	3.3	0.019	−1.76	2.90	0.034	−1.73	3.3	c
544.258	6.08	Glycocholic acid sulphate	M-H	1	0.004	8.29	3.7	0.001	73.7	3.62	0.001	63.2	3.4	b
514.278	6.10	Taurocholic acid	M-H	12	0.031	85.7	6.6	0.013	135.2	5.89	0.012	191.9	5.5	b
624.338	6.22	Glycochenodeoxycholic acid 3-glucuronide	M-H	1	0.034	8.04	2.2	0.018	9.73	1.95	0.020	13.2	1.9	b
464.301	6.23	Glycocholic acid	M-H	1	0.002	32.0	5.3	0.001	40.5	4.78	0.003	39.1	4.5	a
465.249	6.34	Steroid glucuronide (C_25_H_38_O_8_)	M-H	0	0.001	−2.38	2.2	0.004	−2.27	2.14	<0.001	−2.72	2.9	c
528.263	6.76	Glycochenodeoxycholate-N-sulphate	M-H	1	0.006	8.58	7.4	0.001	22.2	6.47	0.003	19.1	6.3	b

CCA, cholangiocarcinoma; HR, high risk; FC, fold change; OV, *opisthorchis viverrini*; PPF, periportal fibrosis; RT, retention time; VIP, variable importance in projection score.

a Level of metabolite identification: (a) identified compound; (b) putatively annotated compound; (c) putatively characterised compound class; and (d) Unknown.

b Negative FC value indicates down-regulated metabolites in cholangiocarcinoma.

c NS = not significant (*p* > 0.05).

## Discussion

The objective of this study was to use MS-based metabolic global profiling and multivariate statistics to investigate whether it is possible to generate biologically meaningful differential urinary metabotype associated with patients with biliary duct tumours. Although the proof-of-principle study was performed with a relatively small number of diseased subjects (*n* = 14), several metabolites of biological interest were discovered from the dataset.

All participants were adult Isaan, living in Northeastern Thailand and at high risk of developing biliary tumours as a result of dietary exposure to *Opisthorchis viverrini*. Unlike CCA in Western countries, where the at-risk population is difficult to characterize, it is possible to investigate urinary metabolic signature during disease development from exposure to cholangiocarcinogenesis in a Thai cohort. Encountered confounding factors (such as gender differences, dietary and environmental influences) were first examined to allow interpretation of genuine biological differences.

As with any adult diseased cohort, drug intake is inevitable and has to be controlled for or taken into account. The analgesic drug, paracetamol, was present in greater concentrations in the CCA urinary metabolome, compared with control groups. Using correlation analysis between the most dominant drug feature and metabolic profiles, it was possible to identify coeluting isotopes, adducts and fragments. To mitigate against these complications, these analytes were all excluded from the analytical models.

Urinary excretion of drug metabolic breakdown end products was identified with reference to previous publications on drug metabolism.^12^ However, it was difficult to study the impact of drugs on endogenous metabolite levels; drugs are metabolised differently from person to person, and without a predose sample, it is difficult to know the impact of drug treatments on metabolic profiles, even after excluding urinary eliminated metabolites of the drug in question. For example, in individuals with increased bacterially mediated p-cresol generation, reduced postdose urinary ratios of paracetamol sulphate to paracetamol glucuronide were identified. The reduced capacity to sulfonate paracetamol results from competitive O-sulphonation of p-cresol.^13^

Tobacco exposure–related biological perturbations were investigated extensively to fingerprint the metabolome of cigarette smokers. A distinct metabolic pattern induced by oxidative stress and cell damage was characteristic of smokers, and these alterations were evident in urinary, serum and salivary metabolic profiles.^14^ Smoking can dominate the urinary metabolome through elimination of tobacco-derived toxins such as nicotine and its metabolites and by altering endogenous metabolism of smokers.

In a study by Garcia-Perez *et al*, capillary electrophoresis–mass spectrometry was applied to profile urine samples of cigarettes smokers and nonsmokers.^14^ Perturbations of glutathione pathway intermediates, such as glycine, cystine and serine, were significantly decreased in smokers, compared with nonsmokers which was hypothesised to be modified by glutathione depletion and oxidative stress resulting from smoke toxicants.^14^ In the present study, no difference was found between smokers compared with nonsmokers after carefully excluding nicotine-related metabolites from the OPLS-DA model.

### Dietary Influences

Exogenously (xenobiotic) and endogenously eliminated metabolites, related to caffeine-containing foods, and possibly medications, were detected in greater concentrations in control groups, compared with patients with CCA. Assessment of caffeine intake in epidemiological study on the US population using LC-MS profiling of spot urine detected several caffeine-related metabolites.^11^ Detection rates of caffeine elimination in urine was over 80% in people who had reported to consume caffeine (from foods, beverages and dietary supplements) in the 24-hr dietary recall interview. Caffeine was mainly excreted as either 1-methylxanthine and 1-methyluric acid or as 3-methylxanthine, 7-methylxanthine and 3,7-dimethylxanthine in the study samples.^11^

In the present study, relative concentrations of several caffeine analytes including 1-methylxanthine, 3-methylxanthine and 1-methyluric acid were highly correlated. Urinary hippurate was also positively correlated with caffeine metabolism products. In addition, urinary hippurate was found to be associated with healthy metabotype and significantly correlated to polyphenols detected in the negative ESI mode. Polyphenol dietary intake, from tea consumption, was shown to be a major source of urinary hippurate and to increase urinary abundance of phenylsulphate substances, including pyrocatechol sulphate, O-methoxycatechol-O-sulphate and vanillin 4-sulphate.^15^ In the liver, hippurate (or benzoylglycine) is synthesised by conjugation of benzoic acid with glycine. Renal clearance of hippurate is influenced by two dietary sources, consumption of foods containing benzoic acid and by degradation of phenolic compounds by gut microbiota.^16^

Tea polyphenols, such as catechins, which are not significantly absorbed in the small intestine, are broken down by colonic microbiota to smaller bioavailable phenolic molecules through bacterial metabolism. Via *β*-oxidation, colonic microbiota converts phenolic degradation products, such as phenylpropionic acids, to benzoic acid which are then eliminated as hippurate.^17^ A healthy diet, containing whole grains, fatty fish, and bilberries, has been shown to increase phenolic metabolites levels (such as hippurate and pyrocatechol sulphate) in blood.^18^

Proline betaine was found significantly (p < 0.001) upregulated in cancer patients' urinary profiles, compared with control groups. This betaine derivative, or trimethylated amino acid, is proposed to play an osmoprotective role for the kidney.^19^ Greater urinary elimination of proline betaine has also been found in patients with other malignant tumours, such as lung cancer^20^ and hepatocellular carcinoma (HCC).^21^ Proline betaine is abundant in citrus fruits and was recently recognised as a putative marker of their dietary consumption.^22^ Without comprehensive dietary intake data available for CCA patients, it is difficult to draw any firm conclusions on this observation.

### Gender Influence on the Metabolic Profile

Variation in the genetic background, physiology, muscle bulk, lifestyle and dietary habits between men and women translate into intrinsic phenotypic differences between the two genders, an issue that has been addressed in several metabonomics studies^,23^. Males showed greater urinary excretion of creatinine (p 0.017) and leucylproline (p 0.014). Higher levels of creatinine in urine were found to be associated with the metabolic signature of male gender, lean individuals and high protein diet^23^ and were directly correlated with muscle mass and moderate/intense physical activity. Leucylproline (a dipeptide formed from the amino acids leucine and proline) loss in the urine is hypothesised to be a product of incomplete breakdown of protein digestion and possibly excreted because of cancer cachexia.^24^

The study participants were mainly farmers from the outskirts of Khon Kaen. In this region, men are typically fishermen, a job requiring muscular strength and high physical activity. They also tend to eat freshly caught fish as it is a convenient and affordable source of protein during the working day. The nature of their job and greater protein intake possibly contributed to this observation between Thai men and women. Urine from females contained higher amounts of two markers of bone collagen degradation, prolylhydroxyproline (p 0.010) and 4-hydroxyproline (p < 0.001). Urinary prolylhydroxyproline was assessed as diagnostic marker of osteoporosis in postmenopausal women and yielded a sensitivity of 74.3% and specificity of 90.8% (area under the curve (AUC) = 0.903) at the cut-off value of 10.2 *μ*mol/mmol of creatinine.^25^

### The Effect of Opisthorchiasis and PPF on the Metabolic Profile

Supervised OPLS-DA models calculated to examine difference between control groups were not predictive. Individuals with opisthorchiasis showed similar metabolic fingerprint to those at risk. OPLS-regression model was trained using fecal egg counts as a continuous outcome variable to identify urinary metabolites associated with liver fluke infection, but it did not produce a positive result. This was expected because of a number of confounding factors: (1) the at-risk group may potentially harbour *O. viverrini*; at a low worm burden (*<* 20 worms); (2) egg counts of opisthorchiasis participants ranged between 6 and 880 eggs per gram of feces, indicating a light to moderate infection with a worm burden of *<*15 to 62 worms, whereas the worm load detected in severe cases reached greater number (up to 651 worms producing 50,000 eggs); (3) the number of *O. viverrini* eggs detected in feces do not necessarily correlate with severity of infection as repeated treatment with the antihelminthic, praziquantel, could falsely impact the results.^26^

Similarly, the multivariate models calculated using spectral profiles of participants with positive ultrasound screening for PPF against the spectral profiles from individuals with negative ultrasonography (the at-risk and opisthorchiasis groups) were nondiscriminant. However, an increase in urinary acylcarnitine excretion was observed in PPF individuals, compared with CCA patients and normal controls in univariate analysis.

### The Metabolic Profile Associated with CCA

Supervised analysis using OPLS-DA was calculated to examine group differences after excluding exogenous metabolic features and xenobiotics attributed to exposure to drugs, caffeine and nicotine from the raw spectral data. Pronounced urinary metabolic signature characterised by perturbations in purine metabolism and alterations in lipid classes including bile acids, acylcarnitines and steroids was associated with patients bearing biliary malignant tumours.

### Purine Metabolism

Purine metabolism has been implicated in carcinogenesis and reported to be altered in the urine biochemical profiles of liver cancer patients.^27^ In CCA patients, urinary elimination of uric acid was found to be significantly upregulated, whereas hypoxanthine and 1-methylhypoxanthine (a hypoxanthine with methyl group) were downregulated, compared with control groups. Uric acid is a renal breakdown product, derived from the oxidation of hypoxanthine by the enzyme xanthine oxidoreductase, an enzyme associated with oxidative stress, cancer aggressiveness and poor clinical outcome.^28^

Murakami *et al* compared the metabolic profiles of resected intrahepatic CCA tumours with their corresponding benign tissues and with tissues from HCC tumours in a Japanese cohort.^26^ Only hypoxanthine and taurine (both overexpressed) allowed intrahepatic CCA to be distinguished from HCC and from adjacent noncancerous tissue.^29^ Interestingly, hypoxanthine phosphoribosyltransferase 1 (HPRT1) was recently identified as an internal reference gene candidate in CCA and adjacent nonneoplastic tissue.^30^

Hypoxanthine-guanine phosphoribosyltransferase (HPRT), enzyme encoded by the HPRT1 gene, is involved in generation of purine nucleotides through the purine salvage pathway.^31^ Elevated hypoxanthine in tissue could result from increase HPRT activity and suggests that purine *de novo* synthesis could have been substituted by the salvage pathway to support proliferative activity in CCA; salvaging the purine ring is preferential as long as hypoxanthine is available for energy conservation and it is more efficient in terms of ATP equivalents.^32^

Urinary 7-methylguanine, a methylated purine, was found in greater quantities in patients with biliary carcinoma. Human exposure to methylating genotoxic agents, such as *N*-nitrosamines (including tobacco-specific nitrosamines), results in the formation of DNA adducts; of which, 7-methylguanine is produced in greatest abundance in various biological specimens such as white blood cells, DNA and urine.^33^ In animal models, the concentration of 7-methylguanine was greater in urine of rats bearing tumours.^34^ Increased 7-methylguanine is possibly linked to dietary *N*-nitrosamine exposure in the Thai population, rather than nicotine smoking as reported by Chao *et al*.^33^ In their experiment, urinary 7-methylguanine was found to be significantly (P < 0.01) upregulated in smokers (4215 *±* 1739 ng/mg creatinine) compared with nonsmokers (3035 *±* 720 ng/mg creatinine).

In this cohort, the relative concentration of 7-methylguanine did not differ between smokers and nonsmokers in each of the control groups, and greatest urinary levels were found in the CCA group, followed by nonsmokers with opisthorchiasis. In the urine metabolome from smokers, cotinine negatively correlated with 7-methylguanine (*r* = −0.04). In addition, associations between 7-methylguanine and nicotine-related analytes were observed only with cotinine. However, although the correlation between the urinary elimination of cotinine and 7-methylgunine was statistically significant (p < 0.05), the correlation coefficient (*r* = 0.37) suggest that the relationship between the two compounds was not biologically related.

### Acylcarnitine Metabolism

Acylcarnitine derivatives were present at different concentrations in the urine of CCA patients, compared with levels reported in the urine of controls, particularly compared with patients with periductal fibrosis. Acylcarnitines are intermediates in the energy metabolic pathway of fatty acid *β*-oxidation, which is a key pathway in tumorigenesis.^35^ They play prominent roles in transporting long-chain fatty acids across the mitochondrial membrane, a tightly regulated system known as the ‘carnitine shuttle’. This involves a multistep process, initiated once fatty acids enter the cellular cytosol and are subsequently activated by esterification to coenzyme A before they can undergo oxidative degradation.^36^

The carnitine shuttle facilitates transport of impermeable long-chain acyl-CoAs into the inner mitochondrial membrane via their corresponding carnitine ester, forming fatty acylcarnitine, a process occurring at the outer mitochondrial membrane where carnitine palmitoyltransferase 1 (CPT1) exchanges the CoA moiety for carnitine. In the intramitochondrial matrix, carnitine palmitoyltransferase 2 (CPT2) converts acylcarnitine back to acyl-CoA, thereby providing substrates for *β*-oxidation, which generate ATP via the tricarboxylic acid (TCA) and respiratory chain.^36^

Perturbation in acylcarnitines has been implicated in various pathological conditions, including cancer. Their profile in malignant disease appears to be specific to tumour phenotypes. In colonic cancer cell lines, the levels of acyl-, isovaleryl-, butyryl- and isobutyryl-carnitine were greater than in ovarian cancer cells. This was also combined by an increase in deoxycarnitine, a carnitine precursor.^37^ The urinary excretion of acylcarnitine in individuals with kidney cancer was found to be grade dependent; acylcarnitines were depleted in early stage, then steadily increased in the urine of patients with grades 2 and 3 tumours. The abundance of carnitine species derived from amino acid catabolism, 2-methylbutyrlycarnitine (C5), hydroxyisovalerylcarnitine (C5–OH), isobutyryl-carnitine (isoC5) and glutaroylcarnitine (C5-DC) and from fatty acid oxidation (C2 and C3) significantly corresponded to tumour grade status in kidney cancer, particularly between grade 1 and the other advanced grades.^38^

Kim *et al*^39^ investigated the metabolic linkage between the urinary metabotype and encoding genes in bladder cancer. The urinary levels of carnitine and several acylcarnitines (isovalerylcarnitine and octenoylcarnitine) were increased, and melatonin, glutarylcarnitine and decanoylcarnitine were decreased in bladder cancer compared with healthy controls. The tissue mRNA expression related to acylcarnitine and carnitine metabolism (CPT1, CPT2, carnitine/acylcarnitine translocase) were downregulated, which the author proposed to result in elevated urinary acylcarnitine excretion from bladder cancer tissue.^39^

In our study, acylcarnitines were mainly depleted in CCA urine samples and considerably increased in presence of biliary fibrosis. In contrast, urinary short- and medium-chain acylcarnitines were significantly increased in HCC patients compared with patients with cirrhosis.^40^ Butyrylcarnitine (C4) and hydantoin-5-propionic acid were identified as potential markers to distinguish between HCC and cirrhosis with AUC of 0.786 and 0.773 for discovery and validation datasets, respectively.

Using serum LC- and gas chromatography (GC)-MS metabolic phenotyping, Bell *et al*^41^ identified increase in several medium- and long-chain acylcarnitines, along with an increase in circulating free fatty acids in cholestatic biliary diseases (primary biliary cholangitis and primary sclerosing cholangitis). They attributed this observation to mitochondrial impairment and to decreased fatty acid oxidation, which have been shown to play a key role in long-term cholestasis.^42^ The serum metabolome characterisation in potentially premalignant cholestatic conditions could provide an insight into changes observed in PPF patients in our study. It is unknown whether in this cohort, patients with PPF were cholestatic, but cholestatic damage is a common biliary tract complication secondary to liver fluke infection.^43^ Thus, in PPF patients, altered hepatic lipid metabolism could be similar to those observed in cholestasis.

An acylcarnitine assay is a promising prognostic approach to detect liver damage induced by *O. viverrini* and may hold diagnostic utility to distinguish between CCA and PPF patients. The concentrations and ratios of acylcarnitine species have been implemented successfully as diagnostic panel for multiple metabolic disorders in newborns. Acylcarnitine patterns, typically in blood, are compared with age-matched reference ranges, and the results are interpreted based on pattern recognition rather than on single abnormal values.^44^

### Cholesterol and its Metabolites

Cholangiocarcinogenesis had a profound impact on the urinary elimination of cholesterol-related lipid classes, bile acids and steroid hormones. Glycine- and taurine-conjugated bile acids were significantly increased in urine from CCA patients compared with all control groups. Studies investigating bile metabolic profiles differentiated between benign biliary disease and CCA, with bile acids significantly elevated in bile from patients with CCA.^45^ Urinary elimination of bile acids is likely to be related to jaundice, which is a common manifestation of CCA and results from an obstruction to bile flow caused by the growing tumour.^46^ Thus, bile acids *per se* are unlikely to be biologically relevant as a biomarker of early tumour activity.

In line with the disturbed metabolism of bile acids, the presence of cancerous bile duct tumours had a profound impact on steroidogenesis. Three glucuronidated steroids in negative ESI mode (cortolone-3-glucuronide, tetrahydroaldosterone-3-glucuronide and 11*β*-hydroxyandrosterone-3-glucuronide) and two unidentified metabolites (*m/z* 465.249 and *m/z* 331.175) with fragmentation pattern of steroids (*m/z* 113, 95, 85, and 75) were depleted in the urine profiles of CCA patients. Urinary steroids have been implicated in hepatic toxicity in animals.^47^

Steroid species were first discovered, in association with CCA, using UPLC-TOF-MS then quantified by GC- and capillary electrophoresis (CE)-MS in rats with induced hepatic toxicity by Kumar *et al*.^47^ They demonstrated decreased urinary concentrations of 11*β*-OH-androsterone, epiandrosterone and oestrone and elevated levels of 11-dehydrocorticosterone following hepatotoxicity. The authors attributed the increase in corticosteroids to stress conditions and the decrease in androgens and oestrogens to liver damage. Global profiling of the urinary metabolic signature in patients with cirrhosis identified a number of significantly depleted steroid glucuronides compared with healthy controls.^48^ The same group further investigated such changes in targeted, quantitative analysis of the steroid metabolome.^49^ A decreased urinary steroid hormone pattern was associated with cirrhotic and early HCC patients. Two steroid markers (epitestosterone and allotetrahydrocortisol) successfully detected early HCC from cirrhosis (AUC = 0.94), compared with the standard serum tumour marker, *α*-fetoprotein (AUC = 0.60). Data obtained prompted the authors to hypothesise that hepatocellular injury, and reduction of hepatic blood flow impaired the liver capacity to biotransform lipophilic substrates via phase I (introduction of functional groups) and phase II (conjugations) to hydrophilic, readily excretable metabolites.

This exploratory study provides proof-of-concept to further pursue development of urinary metabolic markers of CCA. The progression from liver fluke infection to accumulation of scar tissue around the bile ducts was associated with a marked increase in acylcarnitine species. The metabolic shift in presence of malignant biliary tract tumours was more pronounced. Metabolic reprogramming to cope with high energy demand in proliferation was evident in CCA urinary metabolome. The perturbation in purine metabolism intermediates suggests shifting from *de novo* purine synthesis to a more efficient salvage pathway for synthesis of nucleic acids to support CCA genesis. A metabolic switch to promote fatty acid oxidation could explain the depletion of acylcarnitine metabolites in CCA, a mechanistic approach to support cancer cells in hypoxic microenvironment.^50^ Compromised lipid metabolism, in bile acid and steroid homoeostasis, is potentially a reflection of jaundice status and hepatic injury, respectively.

It is worth noting that the identification of biomarker candidates, *per se*, is not problematic. The difficulty lies in the validation and preclinical verification of these putative markers. Future studies should concentrate on validation of these findings. For a better discrimination of active status of opisthorchiasis, urine antigen assays should be considered.

## Conflicts of interest

The authors have none to declare.
